# Functional Haplotype of *LIPC* Induces Triglyceride-Mediated Suppression of HDL-C Levels According to Genome-Wide Association Studies

**DOI:** 10.3390/genes12020148

**Published:** 2021-01-22

**Authors:** Yu-Huang Liao, Leay-Kiaw Er, Semon Wu, Yu-Lin Ko, Ming-Sheng Teng

**Affiliations:** 1 Division of Endocrinology and Metabolism, Department of Internal Medicine, Taipei Tzu Chi Hospital, Buddhist Tzu Chi Medical Foundation, New Taipei City 23142, Taiwan; lyuh5398@gmail.com (Y.-H.L.); erleaykiaw@yahoo.com (L.-K.E.); 2 School of Medicine, Tzu Chi University, Hualien 97004, Taiwan; yulinkotw@yahoo.com.tw; 3 Department of Life Science, Chinese Culture University, Taipei 11114, Taiwan; semonwu@yahoo.com.tw; 4 Department of Research, Taipei Tzu Chi Hospital, Buddhist Tzu Chi Medical Foundation, New Taipei City 23142, Taiwan; 5 The Division of Cardiology, Department of Internal Medicine and Cardiovascular Center, Taipei Tzu Chi Hospital, Buddhist Tzu Chi Medical Foundation, New Taipei City 23142, Taiwan

**Keywords:** hepatic lipase, genome-wide association study, suppression effect, triglyceride, high-density lipoprotein cholesterol

## Abstract

Hepatic lipase (encoded by *LIPC*) is a glycoprotein in the triacylglycerol lipase family and mainly synthesized in and secreted from the liver. Previous studies demonstrated that hepatic lipase is crucial for reverse cholesterol transport and modulating metabolism and the plasma levels of several lipoproteins. This study was conducted to investigate the suppression effect of high-density lipoprotein cholesterol (HDL-C) levels in a genome-wide association study and explore the possible mechanisms linking triglyceride (TG) to *LIPC* variants and HDL-C. Genome-wide association data for TG and HDL-C were available for 4657 Taiwan-biobank participants. The prevalence of haplotypes in the *LIPC* promoter region and their effects were calculated. The cloned constructs of the haplotypes were expressed transiently in HepG2 cells and evaluated in a luciferase reporter assay. Genome-wide association analysis revealed that HDL-C was significantly associated with variations in *LIPC* after adjusting for TG. Three haplotypes (H1: TCG, H2: CTA and H3: CCA) in *LIPC* were identified. H2: CTA was significantly associated with HDL-C levels and H1: TCG suppressed HDL-C levels when a third factor, TG, was included in mediation analysis. The luciferase reporter assay further showed that the H2: CTA haplotype significantly inhibited luciferase activity compared with the H1: TCG haplotype. In conclusion, we identified a suppressive role for TG in the genome-wide association between *LIPC* and HDL-C. A functional haplotype of hepatic lipase may reduce HDL-C levels and is suppressed by TG.

## 1. Introduction

Hepatic lipase (HL, encoded by *LIPC*), a glycoprotein in the triacylglycerol lipase family, which participates in the hydrolysis of triglycerides (TGs) and phospholipids, is mainly synthesized and secreted from the liver [1,2]. It serves as a regulator of plasma high-density lipoprotein (HDL) cholesterol (HDL-C) levels by converting large, TG-rich HDL2 into small, dense HDL3 and facilitates the uptake of chylomicron remnant-like particles [1]. Studies in humans demonstrated that HL deficiency increases the levels of TG-rich HDL2 particles and causes hyperalphalipoproteinemia [2] Thus, HL is crucial for reversing cholesterol transport and plays important roles in modulating the metabolism and serum levels of several lipoproteins [3,4].

Studies in the field of human genetics have been carried out to investigate the genes influencing HDL metabolism and evaluate their relationship with HDL-C levels and the risk of coronary heart disease. Although environmental and physical factors may affect HDL-C levels and HDL function, it has been estimated that 40–60% of the variability in HDL-C levels can be explained by inherited genetic variations [5]. Approaches involving genome-wide association studies (GWAS) have successfully detected common polymorphisms in several known HDL-modulating genes associated with this variable trait through common variant association studies [6]. Nevertheless, few HDL-C variations have been explained by known loci from GWAS, suggesting the existence of additional pathways and targets [7,8,9]. Plasma TGs are routinely measured using lipid profiles and elevated plasma TGs are common clinical findings. The complex nature of this trait correlates with numerous other metabolic perturbations, including depressed HDL-C and directly suggests that TGs cause atherogenesis. TG-elevating variant alleles identified in GWAS generally showed strong associations with clinical cardiovascular endpoints [10]. Nevertheless, the target genes and regulatory mechanisms of HDL-C and TG remain unclear.

Previous GWAS in various ethnic populations showed that the associations between *LIPC* single-nucleotide polymorphisms (SNPs) and lipid levels were mainly significant with HDL-C [11,12,13]. However, findings regarding the association of the *LIPC* promoter SNP C-514 T/rs1800588 with TG and HDL-C levels were inconsistent [14,15]. Numerous studies have been performed to increase the statistical power of the analysis through meta-analyses or phenome-wide association studies. Despite these attempts, with the exception of HDL-C, the association of rs1800588 with diverse lipids was very inconsistent [16,17,18]. Some confounding factors or additional SNPs may exist that are strongly linked with rs1800588 and modify the association with lipid traits.

Mediation analysis is a third variable effect model for investigating causal processes from the effect of the independent variable to the dependent variable [19]. This method has been commonly used in psychological studies [20,21]. To evaluate a hypothesis for mediation, the association between an independent variable and dependent variable is separated into two causal paths. The first path links the independent variable to the dependent variable directly, whereas the second path links the independent variable to the dependent variable through a mediator. A mediated effect indicates that the independent variable gives rise to the mediator, which in turn gives rise to the dependent variable [22]. In the mediational context, the association attenuated by the mediator justifies part or all of the relationship, as it is part of the causal path between the independent and dependent variables. Nevertheless, the magnitude of the association between the independent and dependent variables is likely enhanced by the statistical removal of a mediational effect. Such a change would imply suppression. In a mediation model, a suppression effect exists if the direct and indirect effects of an independent variable have opposing signs [23].

Our previous candidate gene studies involving mediation analysis in a general Taiwanese population provided the first evidence of the suppression effect [24,25]. Recently, we also identified a suppression effect in the association between HL (*LIPC*) variants and HDL-C levels [26], identifying the TG level as a suppressor. This finding suggests that a third factor influences suppression in addition to the genetic variants and several metabolic phenotypes. Additionally, the suppression effect may be crucial and an association may exist between genetic variants and the risk of atherosclerotic cardiovascular disease.

The primary objective of this study was to validate the suppressive effect of mediation analysis in GWAS and identify haplotype blocks in *LIPC* as well as estimate their effect on lipid-proteins, along with HDL-C and TG levels. We also estimated the functional activity of *LIPC* haplotypes to provide further evidence of the presence of genetic factors responsible for the cardiovascular risk associated with lipid profile in Taiwanese populations.

## 2. Materials and Methods

### 2.1. Participants

The study cohort for the GWAS consisted of participants from the Taiwan Biobank (TWB), which gathered information from recruitment centers across Taiwan between 2008 and 2015 [27]. A total of 4675 participants with no history of cancer, stroke, coronary artery disease or systemic disease were recruited and 675 participants were excluded from the analysis according to the following criteria: retraction of informed consent after participation, fasting <6 h and quality control (QC) for the GWAS. Definitions of hypertension, diabetes mellitus, hyperlipidemia and metabolic syndrome are shown in Supplementary Method S1. The Institutional Review Board of Taipei Tzu Chi Hospital (approval number: 08-XD-011), Buddhist Tzu Chi Medical Foundation approved the study. Written informed consent was obtained from all participants before participation.

### 2.2. GWAS Population

The Taiwan Biobank study group designed the TWB genotype array for a high-throughput Affymetrix Axiom genotyping platform. SNPs on the Axiom Genome-Wide CHB 1 Array plate with 5% minor allele frequencies in a set of 4675 samples from Taiwanese population, previously genotyped at the National Center of Genome Medicine of the Academia Sinica, Taipei, Taiwan, were selected for the new TWB array. The genomic DNA was genotyped using the Axiom^TM^-TWB genome-wide array including 642832 SNPs, with the help of the National Center of Genome Medicine of the Academia Sinica. All samples enrolled in the present analysis had a call rate of ≥97%. QC for SNPs was established as follows: SNP call rate <3%, minor allele frequency <0.05 and violation of Hardy–Weinberg equilibrium (*p* value < 10^−6^) were excluded from subsequent analyses. After QC, 4000 participants and 614,821 SNPs were evaluated in the GWAS analysis.

### 2.3. Genomic DNA Extraction and Genotyping

For TWB participants, DNA was isolated from blood samples using a QIAamp DNA blood kit (Qiagen, Hilden, Germany) following the manufacturer’s instructions. SNP genotyping was performed using custom TWB chips and run on the Axiom Genome-Wide Array Plate System (Affymetrix, Santa Clara, CA, USA).

### 2.4. Clinical and Biochemical Analysis

The following clinical phenotypes were analyzed: body height, body weight, body mass index (BMI), waist circumference, waist-to-hip ratio and systolic and diastolic blood pressure. Biochemistry data enrolled for analysis included fasting plasma glucose, as well as lipid profiles including total cholesterol, HDL-C, low-density lipoprotein cholesterol (LDL-C) and TG levels. BMI was calculated as body weight (kg)/height (m^2^).

### 2.5. Haplotype Frequency and Association Analyses

We used Golden Helix SVS Win32 7.3.1 software (Golden Helix, Bozeman, MT, USA) to phase the genotypes and to evaluate the haplotypes of each individual for rs1077834, rs1800588 and rs2070895. The frequency of each haplotype was calculated and only those with frequencies greater than 1% in the cohort were further analyzed. The haplotype association for serum HDL-C levels was tested with linear regression models under the assumption of an additive genetic model and corrected for the effects of age, sex, BMI and smoking, with or without TG.

### 2.6. Mediation Analysis

To explore the mediation effects of TG levels on the association between the *LIPC* haplotype and HDL-C, a mediation model was assumed (Supplementary Figure S1) and the suppression effects were determined by evaluating the following four criteria [24]. Criterion one indicates that the association between the independent variable and mediator must be significant (α). Criterion two indicates that the association between the mediator and dependent variable must be significant after adjusting for the independent variable (β). The calculated product of the two regression coefficients from criterion one and criterion two (αβ) was expressed as the mediation (indirect) effect, which reflected the intermediate pathways from the independent variable to the mediator and in turn to the dependent variable. A direct effect (γ’) was calculated as the regression coefficient connecting the independent variable to the dependent variable when adjusting for the mediator. Criterion three indicated that the association between the independent variable and dependent variable must be significant, which was expressed as the total effect (αβ+γ’). Criterion four indicates that the mediation effect must be significant, which was estimated by using the Sobel test [21,28,29]. Furthermore, a suppression effect could be demonstrated under a condition in which the direct effect was larger than the total effect [30]. Under this condition, direct and indirect effects frequently have analogous magnitudes and opposing signs, which may entirely or partially cancel each other and result in a zero or nonzero but insignificant total effect [31].

### 2.7. Construct

A genomic DNA fragment of the *LIPC* 5′-promoter was amplified by polymerase chain reaction and contained three SNPs, rs1077834, rs1800588 and rs2070895, as reported in our previous association study [26]. The sequences of the forward and reverse primers were 5′-CTCCACGCCCAGCTAACTTTTGTAT-3′ and 5′-GGACTTGTGTCCATTTCTCCGTTTC-3′, respectively. The amplification products were subcloned into the pEASY-T1 vector (Invitrogen, Carlsbad, CA, USA) according to the manufacturer’s protocol. Plasmid DNA was isolated from recombinant colonies and the accuracy was checked by sequencing. The *LIPC* 5′-promoter inserts, which contained the three haplotypes (H1: TCG, H2: CTA and H3: CCA), were subsequently extracted by XhoI and HindIII digestion and gel-purification. The inserts were further subcloned into the pGL4.10 (Luc 2) vector (Promega, Madison, WI, USA), upstream of the firefly luciferase reporter gene. The correctness and direction of the inserts in relation to the luciferase gene were evaluated by sequencing.

### 2.8. Culture of HepG2 Cell Lines

HepG2 cells were cultured in Dulbecco’s modified Eagle’s medium (Sigma, St. Louis, MO, USA) supplemented with 10% fetal calf serum (HyClone, Logan, UT, USA) in 5% CO_2_ at 37 °C.

### 2.9. Transfection and Dual Luciferase Assay

Cells were plated in a 6-well plate and grown to 80–90% confluence. The *LIPC* 5′-promoter constructs (500 ng) were co-transfected with 50 ng pRL-TK (Promega), which encoded Renilla luciferase into HepG2 cells using X-tremeGENE HP DNA Transfection Reagent (Roche, Basel, Switzerland). Luminescence was quantified by using a dual-luciferase reporter assay system (Promega) according to the manufacturer’s protocol after 24 h transfection. All experiments were performed in triplicate and data for firefly luciferase activity were normalized to that of the Renilla luciferase for each sample.

### 2.10. Statistical Analysis

The clinical characteristics of continuous variables are expressed as the means ± standard deviations, which were tested using a two-sample *t*-test or analysis of variance. A chi-square test or chi-square test for the trend was used to examine differences in the distribution of categorical data. TG and HDL-C levels were logarithmically transformed before analysis to adhere to a normality assumption. A generalized linear model was used to analyze TG and HDL-C levels in relation to the predictors of the investigated genotypes and confounders. The genetic effect was considered as additive and adjusted for age, sex, BMI and smoking status. Genome-wide scans were calculated using PLINK. *P* values below the threshold of *p* = 5 × 10^−8^ were considered significant. All calculations were performed using SPSS version 22 (SPSS, Chicago, IL, USA). *P* values <0.05 using a two-sided test were considered significant. Missing data were managed using listwise deletion. The analysis of deviation from the Hardy Weinberg equilibrium, estimation of linkage disequilibrium between polymorphisms, association between haplotypes and lipid-protein levels were performed using the Golden Helix SVS Win32 7.3.1 software (Golden Helix, Bozeman, MT, USA).

## 3. Results

### 3.1. Baseline Characteristics of the TWB Population

Demographic data and lipid profiles of the participants, grouped by sex, are summarized in Table 1. A significantly higher percentage of men was current smokers (*p* < 0.001). In addition, men showed significantly higher BMI (*p* < 0.001), waist circumference (*p* < 0.001), waist/hip ratio (*p* < 0.001) and circulating levels of LDL-C (*p* = 0.006) and TG (*p* < 0.001) than women. In contrast, circulating HDL-C (*p* < 0.001) and total cholesterol levels (*p* = 0.015) were lower in men than in women.

### 3.2. GWAS for HDL-C in the TWB Population

**Figure 1:**
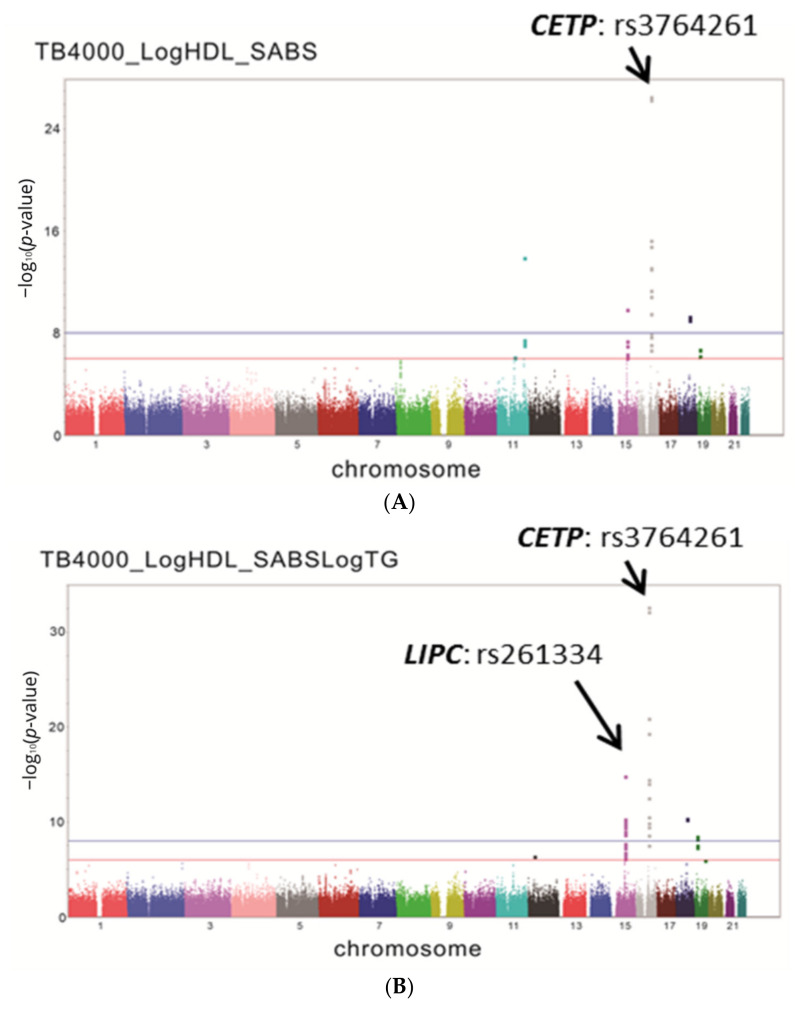

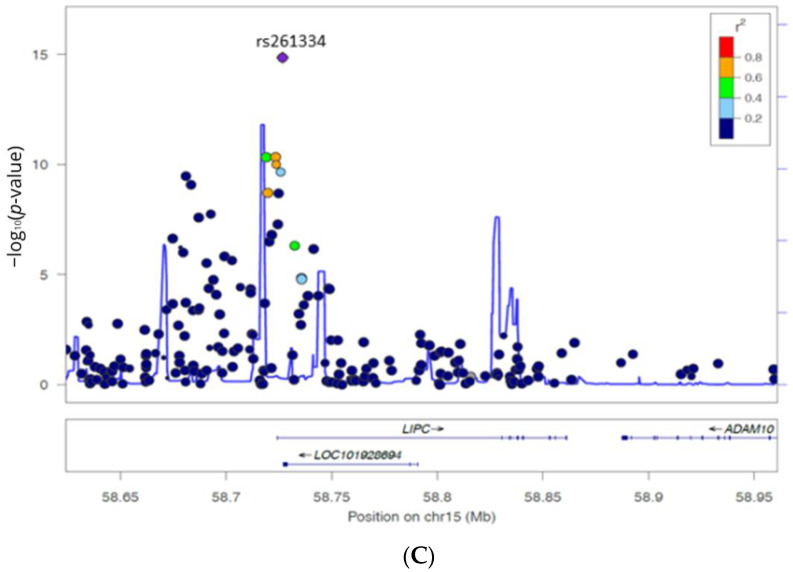
Genome-wide association studies (GWAS) analysis of high-density lipoprotein cholesterol (HDL-C) levels and *LIPC* variants. (**A**) Manhattan plot; (**B**) Manhattan plot after adjustment for TG; (**C**) Genetic region of rs261334 on chromosome 15.

After performing QC following the standard procedure, the results showed that several variants in a region of chromosome 16 had a highly significant association with HDL-C levels. These significant variants were upstream and in the 5′-untranslated region of *CETP* (*p* = 10^−8^–10^−27^) (Figure 1A). After adjusting for TG levels, the region of chromosome 16 still showed a highly significant association with HDL-C levels. Interestingly, the second significant region was in a region of chromosome 15 in *LIPC* (Figure 1B). The most significant SNPs were detected at rs261334, which is located in *LIPC* (*p* = 1.41 × 10^−15^) (Figure 1C). The most striking result was the presence of more than 8 non-coding SNPs in the upstream and 5′-untranslated regions of *LIPC* (Supplementary Table S1), with significant *p* values (*p* < 10^−8^), indicating that the association between *LIPC* variants and HDL-C is strongly suppressed by TG. Linkage disequilibrium analysis showed that rs261334 was in strong linkage disequilibrium (r^2^ = 0.96) with the promoter SNP at rs1800588, which is known to function in regulating *LIPC*. Our candidate gene study also showed that rs1800588 is strongly associated with TG and HDL-C levels. Therefore, rs1800588 was used to replace rs261334 in subsequent analysis.

### 3.3. Association of LIPC Promoter Haplotypes and HDL-C Levels

As single SNP regression analysis revealed that multiple sites within *LIPC* significantly affect lipid levels, haplotypes were evaluated to capture possible allelic associations. The GWAS showed that in the promoter region, in addition to rs1800588 being strongly associated with HDL-C and TG levels, the other two SNPs rs1077834 and rs2070895, located in the transcription factor binding-site, were significantly correlated with HDL-C and TG levels. Therefore, we performed haplotype analysis of these three SNPs (SNP1:rs1077834; SNP2:rs1800588; SNP3:rs2070895). Three major haplotypes were observed, accounting for 99.9% of all inferred haplotypes. After adjusting for age, sex, BMI and smoking, haplotype H2: CTA was found to be associated with significantly increased HDL-C levels (*p* = 0.0295). When further adjusted for TG, haplotypes H1: TCG and H2: CTA were found to be associated with significantly decreased (*p* = 0.0003) and increased (*p* = 0.0009) HDL-C levels, respectively. This indicates that the association of *LIPC* haplotypes with HDL-C was completely or partially mediated by TG (Table 2).

### 3.4. Mediation Analysis of the Association between LIPC Haplotype TCG and HDL-C

Four criteria were evaluated to establish mediation and suppression effects (Table 3). After adjusting for age, sex BMI and smoking, haplotype H1: TCG was found to be significantly associated with lower TG (*p* = 0.025) (criterion 1), which in turn presented significant negative effects on HDL-C (*p* = 9.17 × 10^−48^) (criterion 2). The total effect of H1: TCG on HDL-C was −0.021 (*p* = 0.05) (criterion 3). The results of the Sobel test showed a z value of 2.38 and *p* value of 0.023 (criterion 4). Furthermore, the direct effect (γ’) of H1: TCG on HDL-C (-0.033) was larger than the total effect (αβ+γ’) (0.021) and had the reverse sign with the mediation effect (αβ) (0.012), demonstrating a suppression effect in this model.

### 3.5. Functional Analysis of Three Haplotypes in the LIPC Promoter

**Figure 2:**
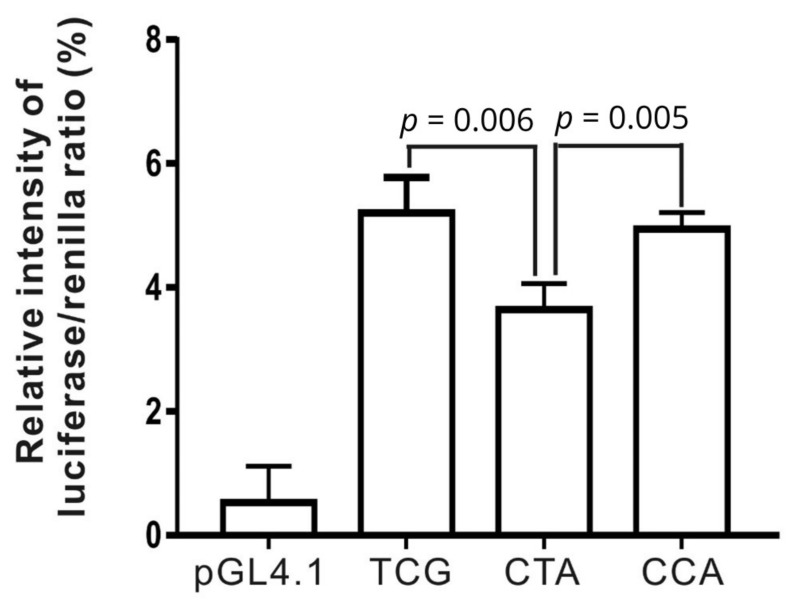
Luciferase reporter assay of pGL4.10-CCA, CTA and TCG in HepG2 cells. Activity of LIPC haplotype H2: CTA significantly decreased 30% and 26.3% as compared with H1: TCG and H3: CCA, respectively. Data were normalized to renilla activity and expressed as percentage of PGL 4.10.

To investigate whether these haplotypes affect promoter activity, they were expressed in HepG2 cells, after which luciferase activity was analyzed in the cell lysates. Compared with the results of the control (pGL4.10 vector), luciferase expression was significantly greater following transfection with haplotypes H3: CCA, H2: CTA and H1: TCG (*p =* 1.8 × 10^−5^, *p =* 0.0003 and *p =* 5.0 × 10^−5^, respectively). In addition, haplotype H2: CTA showed lower luciferase activity than the major haplotype H1: TCG (*p =* 0.006) and rare haplotype H3: CCA (*p* = 0.005), respectively (Figure 2). The results demonstrate that all three promoter haplotypes are functional and that H2: CTA plays a critical role in regulating *LIPC* expression.

## 4. Discussion

This study investigated the suppressive role of TG in a GWAS between *LIPC* and HDL-C. To validate the suppression effect in our previous candidate gene study, we performed a GWAS in a second and larger Taiwanese population. A region in chr.16, which encodes the cholesteryl ester transfer protein (CETP) gene, was found to be strongly associated with HDL-C. This result is consistent with those of previous studies. CETP is an important protein that modulates HDL metabolism and genetic variants in *CETP* have been shown to be associated with HDL-C levels [32]. The protein transfers cholesteryl esters from HDL to apolipoprotein B-containing particles in exchange for TGs, thereby reducing the plasma concentration of HDL-C. CETP inhibitors effectively reduce LDL-C and enhance HDL-C levels [33]. CETP is thus thought to be a target for treatment of increased LDL-C levels and inhibiting atherosclerosis [34,35].

Unlike the consistent results indicating an association between *CETP* and HDL-C, the effect of *LIPC* promoter variants on HDL-C is very variable [36,37,38,39]. Some researchers found no association between *LIPC* polymorphisms and HDL-C [38], whereas others only observed the influence of plasma TGs [39]. Interestingly, we found that several variants in the 5′-non-coding region of *LIPC* were significantly associated with HDL-C only after adjusting for TG. *LIPC* codes for the synthesis of HL, which participates in HDL-mediated reverse cholesterol transport, carrying the TGs and cholesterols from the circulating blood to the liver and is involved in the hydrolysis of TGs [2]. Thus, TGs may have a masking effect on the association between *LIPC* and HDL-C.

To investigate the causality of SNPs in the promoter region of *LIPC*, we identified three haplotypes in the section of the proximal *LIPC* promoter that included three common polymorphisms (-710T/C, -514C/T and -250G/A). Haplotype analysis demonstrated that the common haplotype H1: TCG was significantly associated with TG and mediated the association with HDL-C. However, the minor haplotype H2: CTA lost significant association with TG, though possessing increased significant association with HDL-C. Further, the rare haplotype H3: CCA subsequently recovered the significant association with TG but lost the significant association with HDL-C, irrespective of further adjustments for TG. These findings are comparable to those of previous studies, which reported that the -514T and -250A variants (SNP2 and SNP3) in H2: CTA are associated with elevated HDL-C and TG [38,40,41]. They further reported that both variants are completely linked. However, we found that these two variants combined with -710T/C had the opposite effect on TG and HDL-C levels in H1: TCG and H3: CCA. This observation strongly supports that *LIPC* H2: CTA is the major factor determining plasma HDL-C levels. The effect of the SNP2 C allele may only become apparent in combination with the SNP1 T/C and SNP3 G/A in H1: TCG and H3: CCA.

To explore the possible mechanism of the effect of TG on the association between *LIPC* and HDL-C, we performed mediation analysis to validate the suppression effect. To the best of our knowledge, this is the first evidence of a suppression effect in large-scale GWAS. According to our data, we try to explain the suppression effect more detail by using a pattern with signs and directions (Supplementary Figure S2). As carriers of H1:TCG presented relative lower TG levels when compared with the other haplotypes (upper red arrow; α: −0.053 ± 0.023*), we predicted that the association between *LIPC* haplotype H1: TCG and HDL-C was suppressed by the TG level because of the negative correlation (green circle; β: −0.22 ± 0.014*) between the TG level (upper red arrow) and HDL-C level (right red arrow), which may eventually produce the opposite effect (green arrow; αβ: 0.12 ± 0.005*) to partially reverse the HDL-C level and therefore masks the significance (red square) between *LIPC* haplotype H1: TCG and HDL-C into non-significance (blue square).

The luciferase reporter assay further indicated that the haplotypes were functional and the functional activity of the common haplotype H1: TCG was reduced by the minor haplotype H2: CTA involving the HDL-C increasing alleles -514T and -250A. Several regulatory elements have been reported in the proximal region (from −1600 to +129) of the *LIPC* promoter [42,43,44]. According to the TRANSFAC database [43], the -250 G→A substitution affects the consensus binding sites for c-Myb and CDP. Studies have reported the -514 site to be in the middle of a CAC*GGG sequence, which is very similar to the E-box sequence (CACCGTG) and can bind the upstream stimulatory factors USF1/2 to participate in regulating glucose and lipid metabolism in the liver [45,46]. The -514 C→T substitution has been reported to interrupt the upstream stimulatory factor 1-binding site located in the proximal promoter region of *LIPC* and decrease promoter activity [46]. These results indicate that H2: CTA is unique and alterations in the major influence of TG and HDL-C in H1: TCG may occur through regulatory elements within the promoter region of −250 to −710, suggesting that the proteins binding to these loci interact with each other.

The suppression effect is seldom applied in genetic associations. In the present study, we replicated the suppression effect in a second independent Taiwanese population. By combining the results of haplotype analysis and functional activity, we hypothesized that TG levels mediate the suppression of the association between *LIPC* variants and HDL-C. This suppression effect is due to the multiple functions of HL and CETP in lipid metabolism. HL mainly participates in the hydrolysis of TG from intermediate-density lipoproteins and not from very low-density lipoprotein particles [47,48] and reduces TG levels in the bloodstream. CETP mediates the transfer of TG from the pool of TG-rich lipoproteins to HDL and of cholesteryl ester from HDL to TG-rich lipoproteins [32]. The TG enrichment of HDL enhances the clearance of HDL apo A-I and HDL-C from circulation. Although we demonstrated a possible mechanism of *LIPC* regulation, it remains very important to establish an animal model to confirm this suppressive effect in vivo.

One limitation of our study is that we did not measure HL activity. We predicted that the haplotypes affect the activity of HL and thereby influence plasma HDL-C. Another limitation is that several studies have demonstrated the absence of mutations in the coding sequence of HL that would account for variance in HL activity [40,49,50]. However, no studies have excluded mutations in intronic or distal enhance elements. Therefore, in future studies, *trans*- or *cis*-regulation in the distal region should be considered.

## 5. Conclusions

In conclusion, we provide the first evidence of the suppressive role of TG in the genome-wide association between *LIPC* and HDL-C. We further found that the *LIPC* haplotype is functional. Therefore, the reduced HL activity observed in carriers of H2: CTA may occur because of decreased transcription. This molecular mechanism of gene regulation in the lipid profile should be further analyzed.

## Figures and Tables

**Table 1:** Demographic characteristics of the Taiwan Biobank (TWB) population.

	Total	Men	Women	*p*-Value
Number	4000	1734	2266	
Age (years)	48 (39–57)	47 (38–57)	48 (40–56)	0.558
BMI (kg/m^2^)	24.1 ± 3.5	25.1 ± 3.4	23.4 ± 3.4	<0.001
Waist circumference (cm)	83 (77–89.6)	87 (82-92.9)	79.5 (74–86)	<0.001
Waist-to-hip ratio	0.87 (0.82-0.91)	0.89 (0.86-0.93)	0.84 (0.8-0.89)	<0.001
Current smokers (%)	693 (17.3%)	570 (32.9%)	123 (5.4%)	<0.001
Total cholesterol (mg/dL)	194.4 ± 35.5	192.8 ± 34.4	195.6 ± 36.3	0.015
LDL-C (mg/dL)	121.2 ± 31.9	122.7 ± 31.6	120.0 ± 32.1	0.006
HDL-C (mg/dL)	53 (45–63)	48 (41–55)	58 (49–67)	<0.001
TG (mg/dL)	94 (66–136)	111 (77–164)	82.5 (60–118)	<0.001

BMI, body mass index; HDL-C, high-density lipoprotein cholesterol; LDL-C, low-density lipoprotein cholesterol; TG, triglycerides. Data are presented as means ± SD, percentage or median (interquartile range) as appropriate.

**Table 2:** Association of *LIPC* haplotypes with HDL-C level.

	SNP1	SNP2	SNP3	Frequency	TG		HDL-C		*p1* Value
					Coefficient	*p*	Coefficient	*p*	
H1	T	C	G	0.613	−0.05	0.025	−0.021	0.05	0.0003
H2	C	T	A	0.361	0.01	0.161	0.023	0.03	0.0009
H3	C	C	A	0.025	0.16	0.016	−0.01	0.8	0.28

SNP1: rs1077834, SNP2: rs1800588, SNP3: rs2070895. *p*: adjusted for age, sex, BMI and smoking; *p1*: adjusted for age, sex, BMI, smoking and TG.

**Table 3:** Mediation tests of triglyceride (TG) levels for the association between *LIPC* haplotype and HDL-C levels.

		H1-TCG
**Criterion 1**	α	
	regression coefficient	−0.053
	Standard error	0.023
	*p* value	0.025
**Criterion 2**	β	
	regression coefficient	−0.22
	Standard error	0.014
	^#^*p* value	9.17 × 10^−48^
	γ′	
	regression coefficient	−0.033
	Standard error	0.009
	* *p* value	0.0003
**Criterion 3**	αβ + γ′	
	regression coefficient	−0.021
	Standard error	0.01
	*p* value	0.05
**Criterion 4**	αβ	
	regression coefficient	0.012
	Standard error	0.005
	*p* value (Sobel test)	0.023

α: unstandardized coefficient for the association between *LIPC* haplotype and TG levels; β: unstandardized coefficient for the association between TG and HDL-C levels (adjusted for *LIPC* haplotype) Direct effect = γ′; Total effect = αβ+γ′; Mediation (indirect) effect = αβ; *p*: adjusted for age, sex, BMI, current smoke; *: indicated the *p* value of association between haplotype and HDL-C after adjustment for age, sex, BMI, current smoke and TG; ^#^: indicated the *p* value of association between TG and HDL-C after adjustment for age, sex, BMI, current smoke and haplotype.

## Supplementary Materials

The following are available online at https://www.mdpi.com/2073-4425/12/2/148/s1 Supplementary Method S1: Definitions of hypertension, diabetes mellitus, obesity, current smoking and metabolic syndrome, Figure S1: A conceptual model for mediation analysis, Table S1: Genome-wide association study for HDL-C in the TWB population after adjustment for TG, Figure S2: Suppression effect of TG.
