# Supplementary material for: Functional Haplotype of LIPC Induces Triglyceride-Mediated Suppression of HDL-C Levels According to Genome-Wide Association Studies
Source: Genes (Basel). 2021 Jan 22;12(2):148. doi: 10.3390/genes12020148 (PMC7910859; doi:10.3390/genes12020148)
Supplement: Supplementary file 1 [file genes-12-00148-s001.pdf]

Supplementary method 1.

Definitions of hypertension, diabetes mellitus, obesity, current smoking, and metabolic syndrome

Hypertension was defined as a systolic blood pressure (BP) of ≥140 mmHg and/or a diastolic BP of ≥90 mmHg or a self-reported history of hypertension. Diabetes mellitus (DM) was defined as a fasting plasma glucose level ≥ 126 mg/dL, a glycohemoglobin (HbA1C) value ≥ 6.5%, or a self-reported history of DM. Hyperlipidemia was defined as total cholesterol levels of > 200 mg/dL, triglyceride levels of > 200 mg/dL or a self-reported history of hyperlipidemia. Obesity was defined if body mass index (BMI) was 25 kg/m^2 or more. Current smoking was defined as those who smoked cigarettes regularly at the time of survey. Participants with 3 or more of the following attributes are defined as having metabolic syndrome: (1) BP of at least 130/85 mm Hg and/or with past history of hypertension; (2) triglycerides level of at least 150 mg/dL; (3) high-density lipoprotein cholesterol (HDL-C) level of less than 40 mg/dL for men and less than 50 mg/dL for women or with past history of hyperlipidemia; (4) fasting plasma glucose of at least 100 mg/dL and/or with past history of DM; and, (5) waist circumference greater than 90 cm for men and greater than 80 cm for women.

Supplementary Figure 1. A conceptual model for mediation analysis

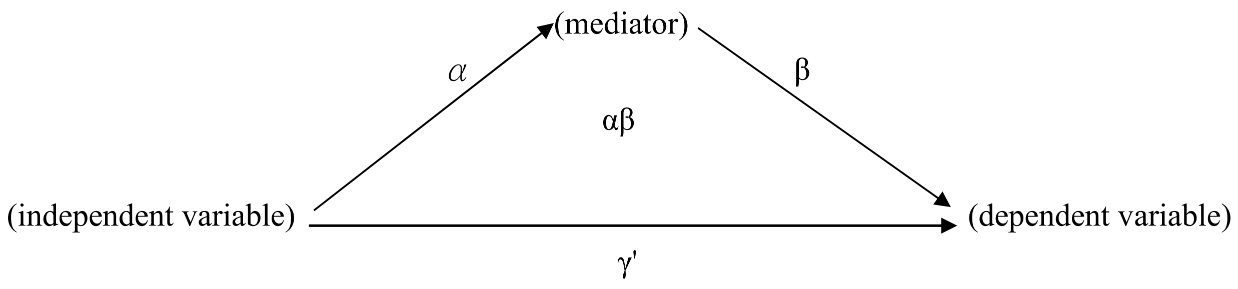

α : unstandardized coefficient for the association between independent variable and mediator.

β : unstandardized coefficient for the association between mediator and dependent variable (when adjusting for independent variable.)

Mediation (indirect) effect = $\alpha\beta$

Direct effect = γ'

Total effect = $\alpha\beta + \gamma'$

Supplementary Table 1. Genome-wide association study for HDL-C in the TWB population after adjustment for TG

CHR	SNP	BP	A1	BETA	STAT	*p*	BETA	STAT	*p1*
16	rs3764261	56993324	A	0.03193	10.92	2.57E-27	0.03208	12.17	2.14E-33
16	rs247617	56990716	A	0.03186	10.87	4.52E-27	0.03193	12.08	6.15E-33
16	rs173539	56988044	T	0.02132	8.156	4.79E-16	0.02177	9.231	4.52E-20
16	rs4783961	56994894	A	0.02081	8.021	1.43E-15	0.02253	9.634	1.07E-21
16	rs11508026	56999328	T	0.0181	7.527	6.58E-14	0.01722	7.919	3.19E-15
16	rs1532624	57005479	A	0.01832	7.497	8.23E-14	0.01726	7.799	8.17E-15
16	rs711752	56996211	A	0.01576	6.951	4.30E-12	0.01503	7.329	2.87E-13
16	rs7499892	57006590	T	-0.01984	-6.799	1.24E-11	-0.01937	-7.341	2.62E-13
16	rs9939224	57002732	T	-0.02005	-6.329	2.79E-10	-0.01913	-6.677	2.82E-11
16	rs1800775	56995236	C				-0.01287	-6.452	1.26E-10
16	rs1864163	56997233	A				-0.0186	-6.325	2.85E-10
16	rs1800774	57015545	T				-0.01721	-5.984	2.39E-09
15	rs261334	58726744	G	0.01471	6.441	1.35E-10	0.01655	8.022	1.41E-15
15	rs7170361	58718998	C	0.01192	5.497	4.13E-08	0.01293	6.603	4.65E-11
15	rs633695	58725839	A	-0.01219	-5.35	9.38E-08	-0.01309	-6.361	2.27E-10
15	rs1077834	58723479	C	0.01127	5.077	4.04E-07	0.01325	6.609	4.47E-11
15	rs2043085	58680954	T	0.01078	4.996	6.14E-07	0.01228	6.293	3.50E-10
15	rs1532085	58683366	A	0.0108	4.977	6.77E-07	0.01206	6.151	8.58E-10
15	rs11635491	58719741	A	0.01119	4.957	7.50E-07	0.01227	6.015	1.98E-09
15	rs8033940	58724842	G	-0.01143	-4.809	1.58E-06	-0.01289	-6.007	2.09E-09
15	rs1800588	58723675	T	0.01068	4.717	2.48E-06	0.01325	6.487	1.00E-10

CHR, chromosome; SNP, single nucleotide polymorphism; BP, base position; BETA, beta coefficient; *p*: adjusted for age, sex, BMI, and smoking; *p1*: adjusted for age, sex, BMI, smoking, and TG

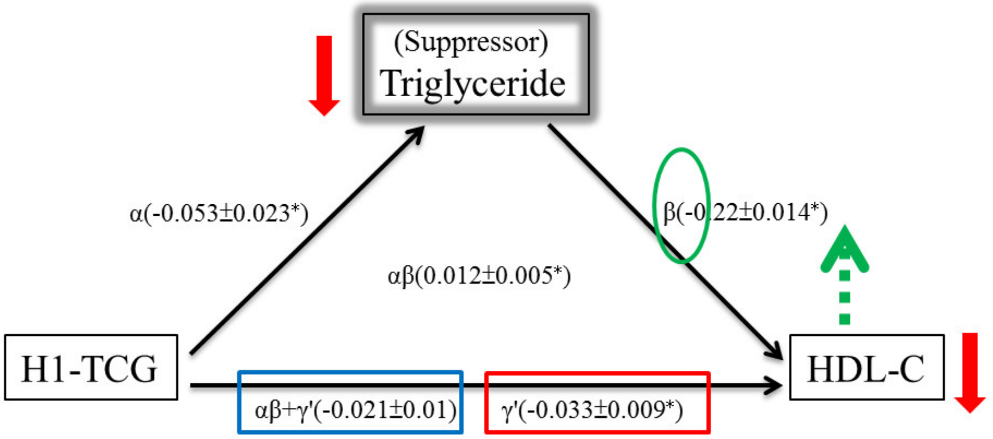

Supplementary Figure 2. Suppression effect of TG

A three-variable mediation model: TG level as a mediator of association between *LIPC* haplotype and HDL-C. Linear or binary regression models were used to assess the path associations of α, β, γ', and $\alpha\beta+\gamma'$
